# Effects of Freeze–Thaw Cycles on Uptake Preferences of Plants for Nutrient: A Review

**DOI:** 10.3390/plants14071122

**Published:** 2025-04-04

**Authors:** Fang Liu, Wei Zhang, Siqi Li

**Affiliations:** 1 Key Laboratory of Mountain Surface Processes and Ecological Regulation, Institute of Mountain Hazards and Environment, Chinese Academy of Sciences, Chengdu 610299, China; liufang@imde.ac.cn; 2 University of Chinese Academy of Sciences, Beijing 100049, China; 3 Key Laboratory of Atmospheric Environment and Extreme Meteorology, Institute of Atmospheric Physics, Chinese Academy of Sciences, Beijing 100029, China; zhangwei87@mail.iap.ac.cn; 4 Qilu Zhongke Institute of Carbon Neutrality, Jinan 250100, China; 5 State Environmental Protection Key Laboratory of Formation and Prevention of Urban Air Pollution Complex, Ministry of Ecology and Environment, Shanghai Academy of Environment Sciences, Shanghai 200233, China

**Keywords:** soil available nitrogen, phosphorus, physiological mechanism, rhizosphere microorganisms, nutrient elements

## Abstract

Freeze–thawing is an abiotic climatic force prevalent at mid-to-high latitudes or high altitudes, significantly impacting ecosystem nitrogen (N) and phosphorus (P) cycling, which is receiving increasing attention due to ongoing global warming. The N and P nutrients are essential for plant growth and development, and the uptake and utilization of these nutrients by plants are closely linked to external environmental conditions. Additionally, the availability of N and P nutrients influences the ecological adaptability of plants. Adapting plants to diverse external environments for the efficient uptake and utilization of N and P nutrients represents a main focus in contemporary ecological research on plant nutrient utilization in the ecosystems of mid-to-high latitudes or high altitudes. Through a comprehensive analysis of the experimental results regarding plant nutrient uptake and utilization in mid-to-high-latitude or high-altitude ecosystems, this paper discussed the processes of soil N and P cycling and the different utilization strategies of nutrient forms employed by plants during freezing and thawing. Freeze–thaw cycles affect the availability of N and P in the soil. Under freeze–thaw conditions, plants preferentially take up readily available N sources (e.g., nitrate (NO_3_^−^-N) or ammonium (NH_4_^+^-N)) and adjust their root growth and timing of N uptake, developing specific physiological and biochemical adaptations to meet their growth needs. When nutrient conditions are poor or N sources are limited, plants may rely more on low-molecular-weight organic nitrogen (e.g., amino acids) as N sources. Plants adapt to changes in their environment by adjusting root growth, making changes in root secretions, and utilizing microbial communities associated with the P cycle to support more efficient P utilization. Future research should (i) enhance the monitoring of plant roots and nutrient dynamics in the subterranean layers of the soil; (ii) incorporate a broader range of nutrients; (iii) examine specific freeze–thaw landscape types, along with the spatial and temporal heterogeneity of climate change within seasons, which is essential for minimizing uncertainty in our understanding of plant nutrient utilization strategies.

## 1. Introduction

Freezing and thawing of soils are physical processes that occur when soil temperature fluctuates above and below 0 °C and are common natural phenomena at high altitudes, high latitudes, or in certain temperate regions [1,2]. The intensity and frequency of soil freezing and thawing primarily depend on local climatic conditions, the thickness of the snow cover, and the soil characterization [3]. As the snow layer covered on the soil surface insulates the soil from the air, it acts as an insulator to some degree, resulting in a reduced variation range in soil temperatures [4]. However, the IPCC report states that global soil surface temperatures are expected to rise by 1.1 °C to 6.4 °C by the end of the century and that winter warming will outpace summer warming [5]. Climate change during winter not only results in significant changes to temperature fluctuations during the freeze–thaw period but also diminishes the thickness of the snowpack and the duration of snow cover [6]. Soil that is deprived of snow cover protection is directly influenced by temperature fluctuations, leading to more frequent occurrences of freeze–thaw cycles. Consequently, it is expected that future widespread freeze–thaw events will be associated with a warmer climate and that the reduction in winter snowpack will result in more intense freeze–thaw cycles in these regions than in previous years [7]. Of course, soil characteristics such as soil texture and moisture content also affect the freezing and thawing process of the soil. Studies have shown that highly connected soil pores promote water migration, accelerate ice crystal formation at freezing fronts, and enhance freeze–thaw damage [8]. Ice crystals expand during freezing in soils with high water content, compressing soil particles, destroying the structure, and increasing freeze–thaw strength [9]. Alterations in freeze–thaw cycling patterns may significantly perturb soil physicochemical properties [10], microbial communities [11], and nutrient dynamics [12,13,14], which will ultimately impact plant adaptation to soil freeze–thaw conditions.

The dry/wet alternation induced by the freeze–thaw cycle can lead to water stress in plants and induce xylem embolism, which subsequently affects plant hydraulics [15] and inevitably impacts nutrient utilization by plants. This water stress induced by the alternating dry and wet conditions does not alter the absolute amount (number of moles) of nutrients in the soil, whereas it does affect the concentration of nutrients within the soil [16]. Most nutrients utilized by plants originate from microbial decomposition products of soil organic matter [17]; meanwhile, a competitive relationship exists between plants and microbes for nutrient utilization. This competition prompts plants to secrete toxic compounds to diminish the unfavorable microbiota, thereby increasing nutrient acquisition during the strong alternating wet and dry processes induced by the freeze–thaw cycle [18]. Moreover, the occurrence of the freeze–thaw cycle may provide enhanced survival opportunities for soil pathogens, resulting in delayed plant seedling phenology and decreased survival probabilities [19]. It was shown that such phenomena of plant low-temperature domestication and genetic variation in response to freezing environments do exist under simulated freeze–thaw test conditions [20]. The freeze–thaw cycle process contributes to varying degrees of increase in the concentrations of soil-dissolved organic carbon/N and inorganic N, which also accelerates the turnover rates of plant fine roots [21]. Soil nutrient effectiveness is a key determinant of plant distribution patterns in terrestrial ecosystems [22], so plants are increasingly diversifying their nutrient acquisition strategies to adapt to the limits of soil nutrient availability [23]. Plants adapt to the changes in environmental conditions by altering their nutrient utilization preferences to survive the varying external stresses, thereby exhibiting differences in plasticity [24]. In comparison to other seasons, the freeze–thaw cycle in winter may represent a particularly critical period for plants facing the stresses from external environmental conditions [25], which typically induces a long-term physiological adaptation [26]. Furthermore, the inherent localized adaptations of plants may serve as a valuable survival strategy for plants in response to global warming [27]. Winter freeze–thaw cycles can alter nutrient availability in the soil [28] while simultaneously enhancing the nutrient flow between plants and soils [29].

The studies about currently known strategies for plant nutrient utilization are predominantly conducted in harsh environments, such as infertile soils [23]. In extremely cold Arctic areas, where the environmental conditions often limit plant adaptation and evolution, plants conserve nutrients in their stems through internal recycling and are motivated to develop appropriate survival strategies in response to environmental stress through physiological self-adjustment [30]. However, below-ground conditions of soils for plants experiencing freeze–thaw cycle processes are likely to be more complex than the above-ground environmental conditions. Moreover, more intense freeze–thaw cycles may result in harsher and more intricate below-ground environments, which poses increasing challenges for studies about plant nutrient uptake. However, within the existing body of literature, relevant reports on plant nutrient utilization preferences are scarce, and even fewer studies address the impact of freeze–thaw cycles on these preferences. In this study, we consider these phenomena of changes in nutrient uptake by plants under specific stress conditions (e.g., freeze–thaw cycle stress in this paper), or weakening or strengthening of nutrient dependence under different stress conditions, as nutrient utilization preferences of plants. Given that soil N and P are key elements limiting plant growth and nutrient utilization strategies and their regulatory mechanisms in plant growth and metabolism within the alpine ecosystems remain unclear [31], this paper reviewed the published research advances in plant utilization preference strategies for these two essential nutrient elements under freeze–thaw conditions.

It is supposed that for the review, a Systematic Literature Review (SLR) method was used to search and select the literature commented on in the paper. Peer-reviewed journal articles reporting the application of ^15^N tracer methods to investigate plant N uptake strategies were identified by entering the following keywords in Google Scholar, the ISI Web of Knowledge, and the China Knowledge Resource Integrated Database: ”nitrogen uptake”, “nitrogen preference”, “nitrogen strategy”, and “stable nitrogen isotope”. To increase comment comparability, the following criteria were applied to select appropriate observations from the initial set of articles retrieved: (i) studies containing ^15^N uptake rates and/or the relative contribution of N forms to plant N nutrients by the ^15^N labeling method were selected; (ii) only the data from experiments with short isotopic exposure times were included; (iii) studies focusing on species-level N uptake but not on community-level N uptake were included. From the screened literature, we found that there are very few studies or reviews on cold ecosystems. Therefore, the question of plant nutrient uptake strategies in cold ecosystems remains open. Our objectives were to review and summarize (i) the characteristics of the processes of soil N and P cycles under freeze–thaw conditions, (ii) plant nutrient utilization strategies during freeze–thaw events, and (iii) knowledge gaps and identify the urgent needs for future investigations. At this point, we expected to provide ecological insights into the underlying mechanisms between soil nutrient cycling processes and plant nutrient utilization strategies under the freeze–thaw cycles in alpine ecosystems.

## 2. Soil N and P Cycling Under the Freeze–Thaw Cycle Conditions

**Figure 1:**
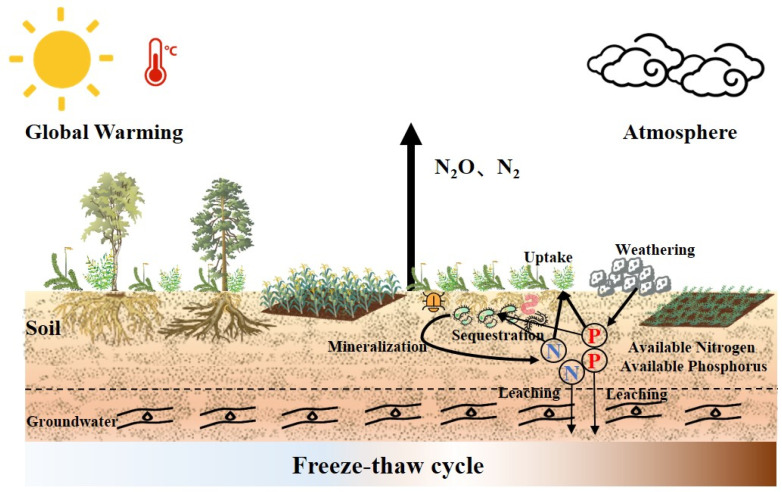
The diagram of nitrogen and phosphorus cycles in soil affected by freeze–thaw events. Nitrogen (N); phosphorus (P).

Soil is located at the intersection of the atmosphere, hydrosphere, biosphere, and lithosphere and involves exposure to their long-term interactions. As one of the key nutrient reservoirs in the ecosystem, the impact of the altered biogeochemical cycling in soil on global ecosystems is significant. Soil N and P cycles are the essential mechanisms that determine ecosystem functions and services, serve as the foundation of soil fertility, and influence the quality or magnitude of ecosystem productivity. Regarding soil N and P cycling processes under the freeze–thaw cycle conditions, a considerable amount of research has been conducted involved with N and P assimilation and mineralization, N-containing greenhouse gas emissions, and N and P losses. These studies primarily focused on the effects of ongoing global warming and the increased atmospheric N deposition on soil N and P cycling processes under the freeze–thaw cycle conditions. Previous studies have focused on N and P cycling in soils during the growing season. In contrast, an increasing number of studies have demonstrated that the non-growing season, which is accompanied by the freeze–thaw processes resulting from the changes in snow cover thickness due to global warming, significantly affects soil N and P cycling [32] (Figure 1). Plant roots and soil microbial activities remain highly active under low temperatures, including freezing conditions ranging from −5 to 0 °C. Approximately 20% to 50% of N and P cycling processes occur during the winter [33,34]. Freeze–thaw processes not only directly affect N and P cycling during the non-growing season, but long-term ecosystem monitoring has also demonstrated that the impacts of the cold winters and frequent freeze–thaw cycles on ecosystems persist into subsequent growing seasons [6,35]. This effect is closely associated with soil properties, microbial activity and community structure, climatic conditions, and vegetation composition [36,37].

In terrestrial ecosystems, frequent freeze–thaw cycle processes promote the increase in unstable N and P in soil, while these nutrients remain capable of participating in in situ nutrient cycling during the deep freezing periods [38]. Nutrients within ecosystems typically exhibit three possible fates to be taken up and utilized by organisms (e.g., microorganisms or plants), to be lost through leaching or to be converted to gaseous forms (e.g., CO_2_, CH_4_, NO, and N_2_O) escaped from the ecosystems [39]. The delivery of soil N to plant roots primarily occurs through mass flow, whereas the delivery of other elements to the root system predominantly occurs via diffusion [40]. N exists in the soil mainly in the form of NO_3_^−^-N and NH_4_^+^-N, both of which are water-soluble ions that readily migrate with soil moisture, driven mainly by water potential gradients [41]. Other elements (e.g., P and potassium) are easily adsorbed by soil particles and form insoluble compounds, resulting in very low concentrations in the soil solution, and diffusion is the main mode of their migration [42]. Since mass flow is proportional to soil moisture, the alternation between dry and wet conditions induced by the freeze–thaw cycles can significantly disturb N transfer [43]. In addition, freeze–thaw cycles lead to the redistribution of soil particles, increasing soil porosity and aeration and thus facilitating water and P transport [44]. Studies have shown that changes in soil carbon balance due to climate warming or human activities are strongly correlated with soil nutrients (N and P), especially in permafrost areas where there is widespread freezing and thawing [45]. The equilibrium between plant nutrient utilization and organic matter decomposition determines, to some extent, whether the soil acts as a carbon source or a sink [46]. Therefore, the determination of the fate of soil N and P within the ecosystem is essential for understanding soil carbon source/sink dynamics. Plants play a pivotal role in the atmospheric carbon balance because their nutrient utilization strategies directly influence the dynamics of N and P in the soil [47]. Climate change influences various aspects of biogeochemical elemental cycling [48,49]. For example, P is primarily affected by geochemical processes such as rock weathering and freeze–thaw cycling, whereas carbon and N are more closely associated with a range of biological processes, including atmospheric N biological fixation and organic matter decomposition [50].

**Figure 2:**
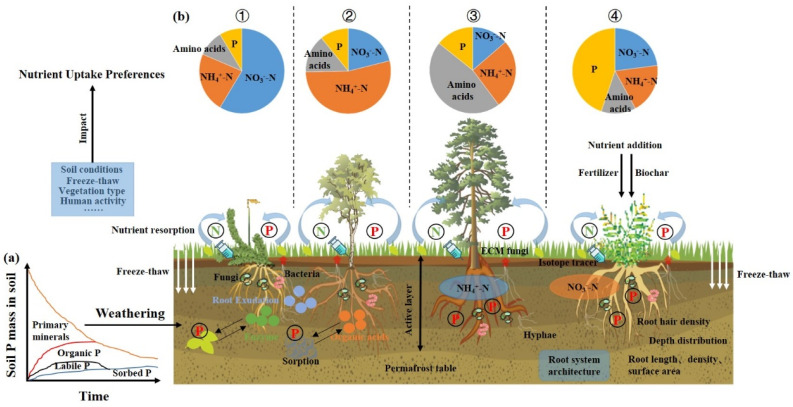
A comprehensive overview of plant nutrient uptake strategies in alpine ecosystems. (**a**) Soil nutrients in response to changes in nutrient availability caused by mineral weathering (P in primary minerals, organic P, labile inorganic P, and sorbed inorganic P). (**b**) Nutrient uptake strategies of plants: ① plant preferences for the uptake and utilization of nitrate nitrogen (NO_3_^−^-N) from the soil; ② plant preferences for the uptake and utilization of ammonium nitrogen (NH_4_^+^-N) from the soil; ③ plant preferences for the uptake and utilization of amino acids from the soil; ④ plant preferences for the uptake and utilization of phosphorus from the soil. Nitrogen (N); phosphorus (P); ectomycorrhizal (ECM).

In summary, the dynamics of soil nitrogen and phosphorus under freeze–thaw conditions directly affect nutrient uptake by vegetation, and this effect is dependent on changes in environmental conditions. This paper offers a thorough discussion of the primary nutrient utilization strategies employed by plants in alpine ecosystems (Figure 2).

## 3. N Utilization Preferences of Plants Under the Freeze–Thaw Cycle Conditions

In most terrestrial ecosystems, carbon is the primary element limiting the growth of soil microorganisms, whereas the primary element limiting plant growth is N [51,52]. Peer-reviewed journal articles reporting the application of ^15^N tracer methods to investigate plant N uptake strategies were identified through searches in Google Scholar, the ISI Web of Knowledge, and the China Knowledge Resource Integrated Database (Table S1). From the 85 papers obtained from the screening, we found that studies from cold ecosystems (Boreal, Alpine, and Arctic) were extremely rare, with only 26 studies (Table 1). Research has demonstrated that the addition of melatonin during the winter wheat season increases the uptake and utilization of nitrate N by the wheat roots whether there is a soil N deficiency or not. This enhancement effect is particularly pronounced in the N-poor environments [53]. Zhou et al. (2017) demonstrated that the incorporation of a soil amendment (i.e., biochar) into soils affected by freeze–thaw cycles enhanced N uptake and utilization by arugula in the subsequent growing season [54]. Notably, the addition of biochar produced at the temperature of 600 °C had the most significant effect on promoting N uptake and utilization by arugula [55]. In northern Alaskan tundra ecosystems, the proportion of NO_3_^−^-N uptake by plants from soils accounts for approximately one-third of the total N uptake. Furthermore, plants in this region have a greater preference for organic-N and NO_3_^−^-N compared to NH_4_^+^-N [56]. However, NO_3_^−^-N is not suggested as the preferred form of N uptake by vascular plants [57].

When plants are at a disadvantage in competition with microorganisms for NH_4_^+^-N [58], they must adjust their N uptake and utilization strategies to alleviate survival stress. For instance, the freeze–thaw cycles can kill the microorganisms to release substantial amounts of organic-N, such as proline and glutamate, which may enhance the preference of uptake and utilization by plants for N derived from microbial lysis [59]. Additionally, the freeze–thaw cycles also enhance the mortality of plant roots; however, plants have a stronger preference for the N from microbial lysis compared to that from plant debris [60]. This preference is potentially associated with the gene expression of plants; for instance, increased expression of *ZmAMTs* genes, which are associated with ammonium transporter proteins, enhances the uptake and utilization of NH_4_^+^-N by plants [61]. However, inorganic-N released from soil mineralization in many freeze–thaw zones often fails to meet the N demands of plants. Additionally, the abundance of organic-N during this period may render the enhancement of *ZmAMTs* gene expression unnecessary for plant N requirements. Plants may change their N utilization strategy from inorganic-N to amino acids, by bypassing the dependence on N produced by the mineralized decomposition, which saves time and reduces the pressure of plant–microbe competition for N [62]. However, not all freeze–thaw areas were demonstrated to be a stronger preference for amino acid utilization by plants. For instance, when NH_4_^+^-N and amino acids labeled with ^15^N were injected in situ into soils in subarctic wilderness areas, plants were found to exhibit stronger and more efficient uptake and utilization of NH_4_^+^-N rather than amino acid [63].

In the middle of the 20th century, influenced by the German scholar Liebig plant mineral nutrition doctrine, formed the core of the traditional nitrogen cycle; that is, plants can only absorb the microbial mineralization of released inorganic nitrogen, such as NH_4_^+^-N and NO_3_^−^-N [64]. In the last 20 years, it has been found that plants can take up low-molecular-weight organic nitrogen (e.g., amino acids, peptides, nucleic acids, and small-molecule proteins) directly from the soil as a source of nitrogen for their growth [64,65,66,67]. During the same period, it was found that two sets of high- and low-affinity transport systems for NH_4_^+^-N, NO_3_^−^-N, and amino acids existed on the surface of plant roots [68], which provided strong evidence for the direct uptake and utilization of low-molecular-weight organic nitrogen by plants from a molecular biology point of view.

Amino acids represent a vital source of N for plants in alpine ecosystems [69]. Research has demonstrated that *Eriophorum vaginatum*, a dominant plant species in Arctic tundra ecosystems, derives approximately 60% of its total nitrogen uptake from amino acids [70]. In contrast, broader investigations into nitrogen acquisition strategies across Arctic tundra flora have revealed substantial interspecific variability, with amino acid contributions ranging from 10% to 82% of total plant nitrogen assimilation. Furthermore, the rate of amino acid, when utilized as a N source, uptake by plants is inversely proportional to the molecular weight of the amino acids [62]. The concentration of free amino acids is typically much higher than that of NH_4_^+^-N following the freeze–thaw cycling. Consequently, plants exhibit reduced potential for amino acid uptake and utilization compared to other N sources, thereby enhancing their efficiency in nutrient acquisition in late spring [71]. The research with the addition of ^15^N and ^13^C-glycine markers to soil microenvironments revealed that plants experiencing freeze–thaw stress reduced their uptake and utilization of glycine, whose extent was correlated to the magnitude of the freeze–thaw cycle [18]. Moreover, following the injections of ^15^N-2-^13^C-glycine into the soils of an evergreen dwarf shrub, a deciduous dwarf shrub (*Salix arenaria*), and a graminoid (*Carex arenaria*), temporal differences were observed in the preferences for glycine uptake and utilization among the three functional types of plants. Specifically, Salix and Gramineae preferentially absorbed more glycine in early winter, whereas evergreen dwarf shrubs exhibited a preference for utilizing glycine in spring [72]. This phenomenon may be attributed to the divergence in temporal ecological niches created by the uptake and utilization of N sources among different functional types of plants [73]. For example, early-growing plants may gain a growth advantage by rapidly taking up N in the spring when temperatures are cooler and soil moisture is plentiful, whereas late-growing plants may begin to grow when resources are more plentiful to minimize competition with early-growing plants for nutrients [74]. Additionally, the experiments with ^15^N-amino acid (glycine) addition in fall involving different functional types of plants in subarctic alpine barrens have demonstrated that the evergreen dwarf shrubs possessed a greater potential for N uptake and utilization in comparison with deciduous dwarf shrubs and graminoids [75]. This may be partially attributed to the enhanced photosynthetic capacity of the evergreen dwarf shrubs in fall [76,77]. However, some studies concluded that the deciduous shrubs possessed the highest capacity for glycine uptake and utilization, followed by the evergreen shrubs, ericoid mycorrhizae, and graminoids [62].

In summary, plants in cold ecosystems have complex and diverse competitive strategies for nitrogen uptake. There is ecological niche differentiation (chemical, spatial, and temporal) in the acquisition of different forms of nitrogen by plants. Changes in soil environment, plant attributes, and biological effects under freeze–thaw conditions may all have an impact on the nitrogen acquisition strategies of plants.

## 4. P Utilization of Plants Under the Freeze–Thaw Cycle Conditions

Although plants are able to utilize other elements (e.g., N, potassium, magnesium, etc.) to a certain extent, P deficiency leads to significant physiological disorders during plant growth and metabolism, which emphasizes the critical position of P in plant nutrition [78]. The soluble phosphates mineralized from the Earth’s crust are the primary P source for plant uptake and utilization, which also is a double-edged sword for P uptake by plants. On the one hand, soluble phosphate meets most of the plant’s P requirements and thus promotes growth. On the other hand, if a plant’s nutrient utilization strategy depends exclusively on a single P source, this strategy is usually unstable and unreliable [79]. It is important to note that both the present and the future may encounter an escalating scarcity of P [80].

Plant uptake and utilization of P show clear environmental adaptations. The supply–demand relationship between P in soils and plants is often characterized by demand exceeding supply. A delayed response to P deficiency occurs in the above-ground tissues when the plant’s below-ground roots struggle to acquire sufficient P to meet its requirement. This delayed phenomenon is attributed to the distance of information transmission between the roots and above-ground tissues [81]. Therefore, to satisfy their P requirements, plants enhance their phenotypes on the one hand and fully utilize microorganisms on the other. When the P levels in soils are low, plants reduce their dependence on mycorrhizal symbionts to strengthen non-mycorrhizal survival strategies, thereby intensifying P uptake and root utilization [82]. For instance, compared to those growing in nutrient-normal habitats, *Acacia mearnsii* growing in nutrient-poor habitats typically secreted higher levels of carboxylates through their roots to improve P uptake and utilization [83]. In terms of the plants’ non-mycorrhizal survival strategies to capture P nutrients, with a greater density of root hairs, they have the potential to capture more P from soil per unit volume. At the same time, due to the lack of P in the soil of the habitat, plant roots will secrete more organic anions (e.g., carboxylates), which will mobilize inter-root P activity, thereby increasing the effective P concentration around the plant roots [79,84,85]. In addition, a molecular-level study of *Arabidopsis thaliana* under the condition of “P starvation” revealed that the *miRNA778* and *SUVH6* genes promoted root growth to enhance P uptake and utilization by increasing gene expression under the condition of “P starvation”. Simultaneously, this study also highlighted the enhancement mechanism of the underground-to-aboveground P nutrient transport under the condition of “P starvation” [86]. The discovery of the over-expressed mechanism of genes facilitated the investigation of the nutrient utilization preferences in plants at the molecular level.

P uptake and utilization strategies in plants are influenced by both the natural environment and human activities. A study conducted in France on turfgrass mulching indicated that P uptake and utilization by plants declined with the decreasing air temperature [87]. Various plant species exhibited differing capabilities to acquire P from soils under low air/soil temperature conditions. For instance, compared to *Trifolium repens*, *Anogeissus leiocarpa* seedlings demonstrated a greater capacity for P absorption and utilization, when they were grown in soils subjected to the frost conditions, and it was observed that soil-soluble P increased post-frost [88]. A study examining soil P dynamics in Arctic tundra ecosystems revealed that the concentration of soil effective P (EEP) remained at a low level (2 µg g^−1^) until mid-July, after which it increased rapidly (5 µg g^−1^) over approximately two weeks. The fluctuation of the EEP concentration was potentially driven by the phased changes in plant P utilization strategies, which occurred at different growth stages to facilitate rapid rooting and development [89]. Furthermore, human activities can also influence plant P utilization strategies. In comparing the contributions of fertilizer additions and plant residue mulching to P uptake by wheat, it was determined that 30% of P uptake by wheat was derived from the plant residues, indicating that, to some extent, residue mulching can reduce the competition for P between plants and soil microorganisms [90]. The absence of livestock grazing in the no-grazing pastures, coupled with the continuous cover of plant residues on the soil surface, can modify plant strategies for P acquisition and mitigate soil P limitations to some extent. The experiments with N and P fertilizer additions conducted on alfalfa at different fertility stages in the semi-arid Loess Plateau region revealed that the P requirements of alfalfa at each stage were significantly higher than N. Furthermore, this study indicated that P fertilizer should be applied when the N/P ratio of the plants exceeds 17 to satisfy the P requirements of the plants; otherwise, P fertilizer addition may adversely affect alfalfa yield [91]. Additionally, it has been demonstrated that the low application rate of P fertilizer under film cover significantly increased the grass yields; however, the medium to high application rate of P fertilizer reduced P uptake and utilization by grass as well as its yields [92].

In summary, the deficiencies in nutrient uptake by plants can be partially compensated by enhancing phenotypic traits, such as root hairs and the primary root, in the subterranean portions of the plant, as well as by maximizing the role of microorganisms [93]. However, it should be emphasized that any factor affecting root secretions and microbial composition significantly impacts P cycling [94]. Phosphorus uptake and utilization strategies of plants under today’s environmental conditions are often influenced by both the natural environment and human activities.

## 5. Envisioning the Future

Plants in alpine ecosystems preferentially utilize nutrients stored in the plant body during the winter; that is, they resort to nutrients in the soil only after the nutrients stored during the overwintering period are exhausted. However, this assumption that plants are dormant in the winter is inaccurate. Despite the air or soil temperatures falling below freezing, vascular plants can still photosynthesize and grow. Therefore, it represents a significant and emerging area of research to study the new mechanisms of soil nutrient transport and plant uptake and utilization from the plant’s perspective within the context of common freeze and thaw cycles that occur in winter. In addition, snow cover will continue to decrease under the climate warming trend, resulting in more intense freeze–thaw cycles in alpine ecosystems that enhance soil mineralization and lead to the accumulation of substantial amounts of active nutrients. These accumulated effective nutrients will inevitably have a significant impact on all stages of plant growth, and quantifying the pulsatile effects of freeze–thaw cycles on the effective nutrients absorbed and utilized by plants will present challenges. We believe that future research can be conceptualized in the following ways:

### 5.1. Enhanced Research on the Subterranean Components of Plants Can Provide a Deeper Understanding of Their Nutrient Utilization Strategies

In the process of ecosystem development, research on the relationship between plant below-ground functional traits and nutrient uptake and utilization is steadily increasing. The root system is a critical component for sensing the soil environment and plays a crucial role in nutrient uptake and utilization. The fibrous roots and pioneer roots of plants perform metabolic and nutrient storage functions, respectively; moreover, pioneer roots exhibit greater tolerance to freeze–thaw stress than fibrous roots. Most plants in the Arctic tundra ecosystem do not exhibit lower below-ground root biomass compared to their aboveground biomass; in terms of average root–crown ratios, the order is sedges > non-grasses > evergreen shrubs > deciduous shrubs. This differentiation in root–crown ratios indicates distinct strategies for growth and nutrient uptake among plant species. Understanding these relationships is essential for addressing the ecological implications of nutrient cycling and species interactions in this sensitive ecosystem. Another summary of 360 studies on nutrients in soil profiles revealed that approximately 90% of these studies collected soil samples only up to a depth of 30 cm, possibly because researchers prioritize nutrients in the upper soil layers, often neglecting deeper soil profiles. The same study, which analyzed 475 root profile datasets globally, indicated that less than 4% of the data volume was sampled beyond a depth of 3 m, potentially because the depth of plant rooting influences sampling thickness. It is important to note, however, that inadequate sampling depth may result in significant gaps in our understanding of the functional characteristics and nutrient conditions of plant roots.

The increased thickness of the active layer and the additional release of nutrients resulting from climate warming may not lead to a genuine increase in plant productivity. This is partly attributable to warming promoting nutrient loss from surface soils as well as the fact that not all plants can utilize the additional soil nutrients; furthermore, plant root densities, depths, and nutrient utilization strategies vary among species. Subsurface ecosystems are predominantly microbial, and the most effective indicators for predicting plant–soil feedback include inter-root community composition and inter-root pathogens (e.g., root-feeding nematodes). For instance, microorganisms regulate P uptake and utilization strategies for plant growth by promoting the mineralization of soil organic P and solubilizing inorganic P. However, the concentration of oxygen influences microbial communities, with fungi serving as the primary agents of decomposition under aerobic conditions, while bacteria dominate under anaerobic or parthenogenetic anaerobic conditions. Most terrestrial plants enhance their ability to utilize nutrients such as N and P by selectively engaging in symbiotic relationships with tufted mycorrhizal fungi or ectomycorrhizal fungi, thereby exhibiting greater plasticity in their nutrient utilization strategies. Conducting quantitative research on below-ground root monitoring, root functioning, and inter-root microbiota presents significant challenges; consequently, thoroughly assessing the relationship between below-ground root functional characteristics and plant nutrient utilization strategies in the context of climate change is notably more complex than evaluating aboveground components.

### 5.2. Incorporate Additional Nutrients into the Study and Examine Their Interrelationships

The biogeochemical processes governing the nutrients essential for plant growth are significantly influenced by climate change, with these nutrients being interrelated. The functional traits exhibited by diverse species, coupled with their distinct survival strategies, result in varying preferences regarding the uptake and utilization of each nutrient. The strategy for carbon utilization in plants, within the context of a warming climate, is expected to remain stable; conversely, the strategies for N and P utilization are contingent upon temperature and precipitation. Specifically, the capacity of plants to absorb and utilize N diminishes as temperatures rise beyond a specific threshold, whereas, in contrast to N, P uptake and utilization by plants are likely to be restricted by precipitation. Most plants preferentially uptake P (>70% of total nutrient uptake) rather than N from senescing leaves for nutrient reabsorption. Within the same natural habitat, manganese concentration in leaves serves as a potential indicator of P uptake and utilization among plant species. Variations in nutrient element preferences among plant species are contingent upon the turnover rate of the nutrient within the plant, while preferences within the same plant species are influenced by soil properties (e.g., nutrient availability and soil texture). To effectively link nutrients to distinct ecological features of biomes, all nutrients must be considered in ecological stoichiometry studies, which should not be restricted to a limited subset of nutrients such as carbon, N, and P.

### 5.3. Revising Research Ideas and Extending the Research Timeline

The presence of a range of uncontrollable and unpredictable environmental factors, such as soil freezing and thawing and extreme weather, coupled with the current limitations in research methodologies and advanced equipment, poses significant challenges for in situ testing. Current research on plant nutrient uptake and utilization predominantly focuses on N, whereas studies related to P tend to emphasize soil erosion. In the context of climate warming, formations such as circles, terraces, and stripes, which arise from freezing and thawing processes, frequently occur in the permafrost zone. These formations effectively illustrate the spatial heterogeneity of plant communities and abiotic conditions, serving as clear indicators of climate change responses, which warrant further investigation in future research.

Plants exhibit thermoplastic leaf movements in response to cold and low temperatures, a physiological adaptation that mitigates excessive photoinhibition, thereby enabling leaves to rapidly recover from winter photoinhibition and optimize nutrient utilization in the spring. Concurrently, winter snowpack, soil temperature, and interactions among herbivores and soil nutrients may influence plant responses to climate change. This relationship underscores the necessity of considering the impacts of climate change on plant nutrient uptake and utilization across seasons in global change experiments.

## 6. Conclusions

Through a comprehensive review of relevant research conducted both domestically and internationally, we have determined that the freeze–thaw cycle will alter plants’ nutrient utilization strategies. This alteration is reflected not only in the quantity of various nutrients absorbed but also in the shifts in the types of nutrients taken up and utilized. The availability of nutrients in the soil will change with climate change. At mid-to-high latitudes or high altitudes, the subsurface environment is likely to be more complex than the aboveground environment; moreover, with the intensification of the freeze–thaw cycling process resulting from the framework of global warming, subsurface environmental conditions are anticipated to become increasingly harsh. As the external environmental conditions change, plants actively adjust their nutrient utilization strategies as an instinctive adaptation to environmental stressors. The study of plant nutrient utilization strategies is integral to understanding ecosystem succession, and climate warming is poised to play a significant role in this process. Identifying effective nutrient gradients and the habitat characteristics that influence plant preferences for nutrient uptake will help us gain a deeper understanding of the ecological mechanisms underlying soil–plant assembly, while also providing a theoretical foundation for new insights into nutrient release, transport, and migration within soil profiles.

## Figures and Tables

**Table 1:** Statistical results of the study of global vegetation nitrogen utilization strategies.

Ecosystem	Climatic Regions	Number of Studies ^1^
Forest	(Sub)Tropical	6
Forest	Temperate	14
Forest	Boreal	2
Grassland	Alpine	13
Grassland	Temperate	12
Tundra	Alpine	2
Tundra	Arctic	4
Other	Other	32

^1^ Statistics based on the published literature.

## Data Availability

No new data were created or analyzed in this study. Data sharing is not applicable in this study.

## Supplementary Materials

The following supporting information can be downloaded at: https://www.mdpi.com/article/10.3390/plants14071122/s1, Table S1: Plant nitrogen uptake preference in natural ecosystems at the global scale.
